# Communication from W. S. Robertson, M.D., Columbus City, Iowa

**Published:** 1859-09

**Authors:** 


### CORRESPONDENCE.

EDITOR OF CHICAGO MEDICAL JOURNAL:

DEAR SIR,—The following cases having come under my notice, are of so unusual a character, that I have concluded to report them (if deemed worthy) through the columns of the "Journal," partly for the sake of obtaining information, and that, should any of your readers meet such cases, they may have the benefit of my observations.

Three cases have occurred in the same family. The first I did not see, but it was described to me by the family physician as "a case of croup, accompanied with pneumonia, attended with some ulceration of the mucous membrane of the mouth and throat." Termination—fatal.

CASE II.—A. E., five years old, was taken ill on Monday night, January 10th. The parents being very much alarmed, and unable to call Dr. C., the family physician, on account of ice in the river, called upon "an eclectic," who pronounced it croup, and, I suppose, treated it accordingly, until Wednesday, when he gave it up, and Dr. C. and I were summoned.

The patient was so much exhausted when we arrived as to give us little hope for the benefit of medical interference. The urgency of the symptoms, however, induced us to administer an emetic, which, acting, afforded temporary relief.

We regret the extreme prostration which prevented a satisfactory examination of the throat, but we could see the ulceration upon the tonsils. The parents informed me that the child had had a sore mouth and throat about a week.

The immediate danger being from catarrhal symptoms, we prescribed:

℞ Comp. syr. squills, ℥j.

Copaiva, ʒss.

Spr. nitre, ʒss.

Ft. expectorans.

Of which we gave half a teaspoonful *pro re nata.*

This patient also died as of croup, on Thursday.

The post mortem appearances—external,—as described by the friends, were: unusual rigor mortis; throat swollen even with the chin and discolored; *knotty appearance* of parietes of abdomen, with discolored patches.

CASE III.—Girl, aged seven years.

At the time of my first visit, my attention was directed by the parents to this child. She was running about the house, but had this same croupous cough; and I was astonished to find already developed, extensive ulceration of the tonsils, of a pearly color and elevated edges.

Ordered solution of nitrate silver, thirty grains to the ounce, applied twice a day to ulcerated surface. Used expectorant as before prepared.

*Friday*, 14*th*. Was called to see her. Found her with high fever, furred tongue and anxious countenance. Cough better. Passing rapidly through this first stage, as it were, we find her vomiting a tough albuminous fluid, afterward serum colored with bile. Frequent alvine discharges from the bowels, resembling meat washing. Intense pain, extending from the throat to the stomach and bowels, causing her to strike herself and cry out in the extremity of her suffering.

While upon symptomatology, allow me to digress so far from this point as to follow out the fatal cases in the third or last stage.

Increase of croupous symptoms; tympanitic distention of abdomen, with intestinal pain, increased by pressure; torpidity of bowels; suppression of urine. Death.

To return to the case in hand.

We find that in each case there has existed evidence of abdominal lesion, as well as the post mortem appearance of the parietes of the abdomen. The inflammation in the throat was apparent; and the intense thirst, tumefaction and tenderness of the bowels, all indicated, as I thought, distinct marks of intestinal derangement, not unlike what we often find in cases where we have inflammation of the mesenteric glands and glands of Peyer. With that view of the case I prescribed—

| | | | |
|---|---|---|---|
| ℞ | Nitrate silver, | grs. viii. | |
| | Liquorice root, pow'd, | " xxiv. | |
| | Syrup, | q. s. | Ft. mass. |

Make 24 pills.

Of these I gave one every three hours. Increased copaiva in expect. to ℥j., and continued as before. This course was continued until Saturday evening (twenty-four hours), when I ordered a portion of castor oil, and with it thirty drops oil turpentine. Operated well, bringing away long shreds of mucus.

*Sunday Morning*. Continued pills. Bowels still rather tympanitic. Ordered—

| | | |
|---|---|---|
| ℞ | Sub. mur. hyd., | grs. vi. |
| | Ipecac., | " vi. |

Ft. chart. vi. Gave one powder every three hours.

9 *o'clock p.m.* Improving. Less tympanitic; less pain. Discontinued pills.

*Monday*, 9 *a.m.* Stop powders; gave two blue pills. Operated.

1 *o'clock*. Discharge more healthy in appearance; patient feels comfortable. Directed wine whey and serpentaria infusion.

Not being able to cross the river, did not hear from her for near a week, when I called, and found her sitting up, and, excepting a little irritation about the fauces, enjoys her usual health. I neglected to state that the solution of muriate silver was continued twice a day to the throat during the course of treatment.

The difference in treatment is obvious. The first cases were treated as for laryngeal disease, while in the latter, comparatively little attention was paid to croupous symptoms, and remedies were administered for an ulcerative inflammation of the alimentary canal.

I confess I am at a loss for a name for the disease. Perhaps some light may be thrown upon the case by stating that there is an hereditary scorbutic taint in the family, although not showing itself in this younger generation before.

Is this a species of scorbutis? a violent form of typoid fever? or, could that inflammation extend, in any way, from the œsophagus to the trachea and produce such symptoms? I think not.

If my communication should be considered worthy a place in your Journal, I would be pleased to hear from some member of the profession upon the subject. Yours truly,

W. S. ROBERTSON.

COLUMBUS CITY, IOWA, Jan. 26th, 1859.

[The three cases detailed by Dr. Robertson present symptoms of diptherite, a disease that has been prevailing in England and America within the last year to a fearful extent.

We congratulate our correspondent on his success in saving even one patient out of the three. The same good fortune has not attended the efforts of others when they had to deal with the disease under conditions marked by the same malignancy of development. We, however, attribute the favorable result in the third case more to the application of nitrate of silver in solution to the fauces, and to its tonic properties when prescribed in pills, than to the purgative action of the castor oil and turpentine.* Neither can the improvement observed in this case at 9 o'clock p.m., on Sunday, the 16th, be fairly attributed to the action of the calomel, considering that it was ordered on that morning, and not more than one grain every third hour prescribed. Turpentine has certainly been recommended by some who have given their attention to the disease. Dr. Perry advises ℨij. of the ol. terebinthinæ to be rubbed up with the yelk of egg and ℥xij. of syrup; one teaspoonful to be given in milk every second hour.

As diptherite is just now exciting much interest, and as our table is covered with the results of the most recent inquiries respecting this disease, we purpose, in the October number, to

* We do not take into account the wine and serpentaria, as they do not seem to have been prescribed until the danger was over.

present our readers with whatever in reference to it is contained in recent publications deserving of notice. At the same time, in giving a summary of opinions gleaned from American and foreign works, we will endeavor to unravel from the confusion a subject which, from its having several points in common with other diseases, appears, at first view, inextricably entangled with them, though really separated by essential differences.]

